# Wheel Alignment of a Suspension Module Unit Using a Laser Module

**DOI:** 10.3390/s20061648

**Published:** 2020-03-16

**Authors:** Seong Han Kim, Kang In Lee

**Affiliations:** 1 School of Intelligent Mechatronics Engineering, Sejong University, 209 Neungdong-ro, Gunja-dong, Gwangjin-gu, Seoul 05006, Korea; shkim8@sejong.ac.kr; 2 R&H Research Lab, Hyundai Motor Company, 150, Hyundaiyeonguso-ro, Namyang-eup, Hwaseong-si, Gyeonggi-do 18280, Korea

**Keywords:** Wheel alignment inspection, Laser module, Vision system for wheel alignment inspection

## Abstract

Vehicle wheel alignment inspection is generally carried out using a computer vision-based system. Due to its inspection mechanism using four wheel centers, the computer vision-based system cannot be applied to the wheel alignment inspection of suspension module units. However, when a vehicle suspension module is being developed, there is no complete car ready for wheel alignment inspection even though it is a very important procedure for suspension property tests. This study proposes a novel and efficient way to inspect vehicle wheel alignment for suspension modules. Two laser modules and several mechanical jigs were employed for wheel alignment inspection, allowing the toe and camber angles of the suspension module to be measured. For accurate wheel alignment results, calibration of the laser modules was performed prior to the inspection. This calibration procedure adjusts the yaw and pitch angles of the laser module so that they can be orthogonal to the mounting jig. For the calibration, a novel method of using laser straightness was adopted and, consequently, 0.02 degrees of orthogonality was achieved. The wheel alignment inspection results were determined then verified using a vision system with two cameras. In order to use this vision system, two cameras were used and a new method of modifying the measurement mechanism was developed. According to the verification results, the proposed wheel alignment inspection provided very high measurement accuracy. The wheel alignment inspection mechanism proposed in this study can not only give very reliable results but also provide a cost-efficient method of inspecting the wheel alignment of suspension modules to automakers.

## 1. Introduction

Vehicle wheel alignment inspection is generally conducted in automobile maintenance shops. It includes the measurement of the steering axis inclination (SAI), caster angle, camber angle, and toe angle. SAI (also known as king pin inclination (KPI)) and caster angle are the inward and backward tilt, respectively, of the suspension toward the center of the vehicle. They are determined by the positions of the upper and lower ball joint pivots, which are geometrically unchanged and thus not adjustable once the vehicle has been designed. Camber angle is the inward or outward tilt of the front tires as viewed from the front, and toe angle is the side-to-side difference in distance between the front and rear of the front tires. These camber and toe angles are adjustable and should be continuously managed by the vehicle owner, because if a vehicle has incorrect camber and toe angles, the vehicle can experience several issues such as uneven tire wear, steering wheel pulling, steering wheel shimmy, and sometimes serious vehicle vibration [1,2].

Wheel alignment inspection in automobile maintenance shops is generally conducted by a computer vison-based system [3,4] comprising specially designed target boards and image acquisition modules, including charged coupled device (CCD) video cameras and infrared light emitting diodes (IR LEDs) as illuminants [5,6]. Wheel alignment inspection using this computer vision-based system needs a full car, which means it needs four wheels for the inspection. The detailed inspection method is described in Section 2.

**Figure 1:**
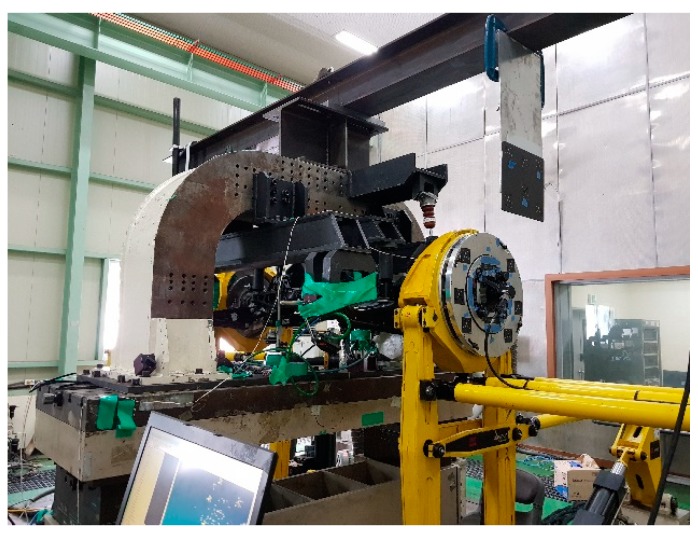
Suspension module under preliminary tests.

When a new vehicle goes into production, its mechanical and electronic modules are manufactured and tested before they are installed in the vehicle. For instance, a suspension module, which is composed of a sub-frame, suspension springs, shock absorbers, lower and upper arms, knuckles, rubber bushes, stabilizer links, and a stabilizer bar, is produced and tested before it is installed into the vehicle. Figure 1 shows a suspension module that is undergoing preliminary tests. The preliminary tests generally include dynamic performance tests, kinematic and compliance (K&C) tests, and durability tests [7,8]. These tests are mainly affected by the geometrical dimensions and mechanical strength of the module parts, and the shape and stiffness of the rubber bushes. In particular, the initial shape deformation of the rubber bushes affects the test results due to preloads and wheel alignment and, accordingly, the preloads on the rubber bushes and the wheel alignment should be thoroughly managed before the tests. Preloads on rubber bushes are usually caused by two factors—suspension module assembly procedures and incorrect wheel alignment [9]. Whereas preloads using assembly procedures can be removed by run-in procedures, preloads by wrong wheel alignment are difficult to remove. Furthermore, when a full vehicle is not yet available, the computer vision-based system widely used in automobile maintenance shops cannot be applied to the wheel alignment inspection because it needs four wheels for the inspection.

There have been several studies that can be applied to the wheel alignment inspection of suspension modules. Dongyoub Baek has proposed a simple and inexpensive method using a consumer-grade depth-sensing camera such as Kinect to replace the computer vision-based system [5]. The method proposed in his study generates point clouds within a region of interest (ROI) that contain the geometrical information of the wheel. He verified the developed method by comparing the wheel alignment results with the results from the existing method. Jieh-Shian Young has proposed a micro control unit (MCU)-based camber inspection system with a 3-axis accelerometer [10]. He emphasizes the fact that the axes of the accelerometer can be misaligned to the axes of the camber inspection system and, therefore, in his study he proposes the calibration method to amend these axis misalignments. Rocco Furferi has proposed a 3D machine vision-based system for the contactless reconstruction of vehicle wheel geometry, with a particular reference to characteristic planes [11]. The effectiveness of the proposed method in his study was verified by comparing the method with the measurement results using a commercial 3D scanner.

This study proposes a new and cost-efficient method to inspect vehicle wheel alignments for suspension modules. The proposed method employs two laser modules that are mounted into the wheel knuckles. With the laser modules, toe and camber angles can be measured by measuring the distance of the laser mark projected on the opposite side of the laser mounting jig. For accurate wheel alignment results, the calibration procedure of the laser module—which makes the laser module perfectly orthogonal to the laser mounting jig—was performed before wheel alignment inspection. After the wheel alignment inspection, the results were verified by a vision system. For the verification, a new method using complementary metal-oxide semiconductor (CMOS) cameras was also developed.

The wheel alignment inspection method proposed in this study can provide very accurate results and a cost-efficient way for automakers to inspect the wheel alignment of suspension modules. In addition, it can be also applied in the wheel alignment inspection of motorcycles and unconventional automobiles [12,13].

## 2. Overview of Typical Wheel Alignment Inspection for a Full Car

**Figure 2:**
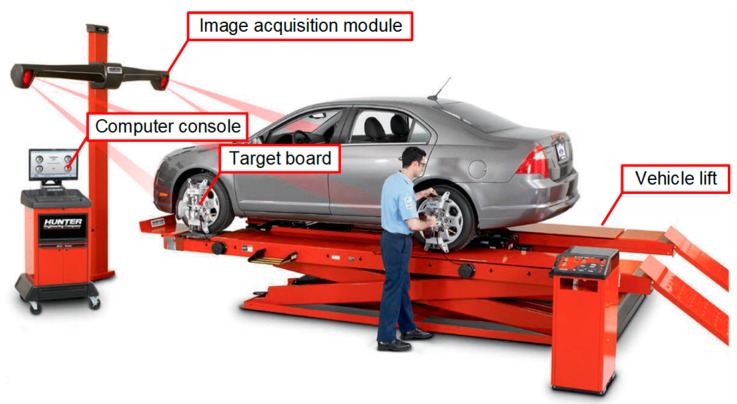
Overall structure of a typical wheel alignment inspection system for a full car [3].

The typical wheel alignment inspection for a full car uses a computer vision-based system [14,15]. Figure 2 shows the overall structure of the typical wheel alignment inspection system, which consists of one vehicle lift with four turnplates, four target boards, image acquisition modules including four pairs of CCD cameras and IR LEDs illuminants, and a computer console with software including various wheel setting specifications. For the wheel alignment inspection of a vehicle, the vehicle is placed on the lift. Each wheel should be positioned on each turnplate of the lift to have four wheels steered freely. Afterward, one target board is attached onto each wheel. The target boards have materials that reflect the illumination from the IR LEDs so the wheel angles (i.e., toe and camber angles) can be measured by the CCD cameras. All wheel alignment values are transmitted to the computer console, which displays the measured values.

**Figure 3:**
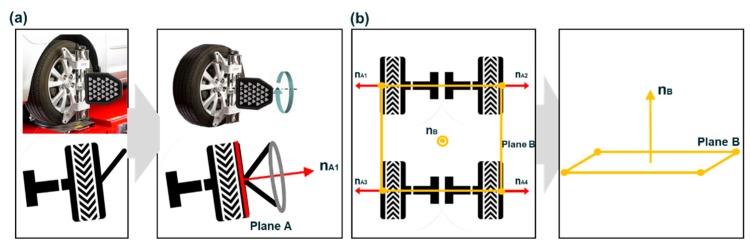
Creation of wheel planes and normal vectors. (**a**) Plane A. (**b**) Plane B.

**Figure 4:**
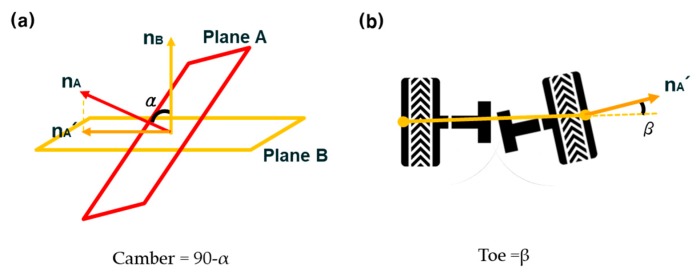
Camber and toe angles from Planes A and B. (**a**) Camber angle. (**b**) Toe angle.

When all target boards are mounted on the wheels, the technician turns the steering wheel to the left and right ends to determine the neutral position of the front wheels. Then, the steering wheel is positioned at the neutral position and the technician moves the vehicle back and forth. By moving the vehicle back and forth, each plane of four wheels (Planes A1, A2, A3, and A4) and their normal vectors (n_A1_, n_A2_, n_A3_, and n_A4_) are obtained, as shown in Figure 3a. Along with the normal vectors, the centers of Planes A1–A4 are also determined, and these centers create another plane (Plane B) and its normal vector (n_B_), as shown in Figure 3b. The typical wheel alignment method using a computer vision-based system uses Plane A and B and their normal vectors to measure the wheel alignment. Figure 4 describes how to measure the camber and toe angles of a vehicle. When there is an angle, α, between n_A_ and n_B_, 90-α is the camber angle. A total of four camber angles can be obtained from each plane of the wheels. On the other hand, when n_A_ is projected onto Plane B, it creates a vector (n_A_’), and the angle between n_A_’ and the centerline of two front wheels is the toe angle of the front wheels. A total of four toe angles can be obtained.

The camber and toe angles of a vehicle are systematically managed by the vehicle’s automaker because they can cause uneven tire wear, steering wheel pulling, steering wheel shimmy, and sometimes serious vehicle vibrations. Camber angles are generally within ±2 degrees, but most modern passenger cars generally have negative camber angles, whereas toe angles relatively have a small angle range of ±0.2 degrees [2,16].

## 3. Wheel Alignment Inspection for Suspension Module

The typical wheel alignment inspection in Section 2 requires four wheels, because the centers of four wheels form a plane and the camber and toe angles are measured with respect to this plane. However, when a suspension module is in development for a new vehicle and its K&C characteristics need to be tested for performance evaluation purposes, the typical wheel alignment inspection cannot be applied because the suspension module has only two wheels. Therefore, a new method of inspecting the wheel alignment is needed for single suspension modules.

### 3.1. Wheel Alignment Inspection Mechanism

**Figure 5:**
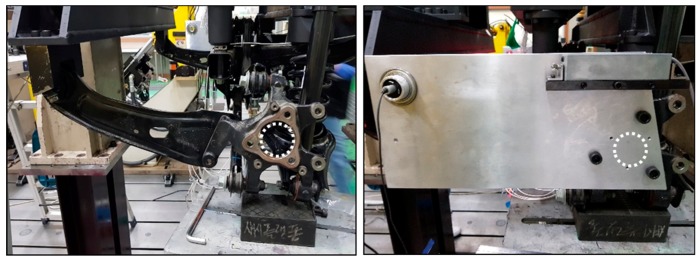
Wheel knuckle and mounted jig.

**Figure 6:**
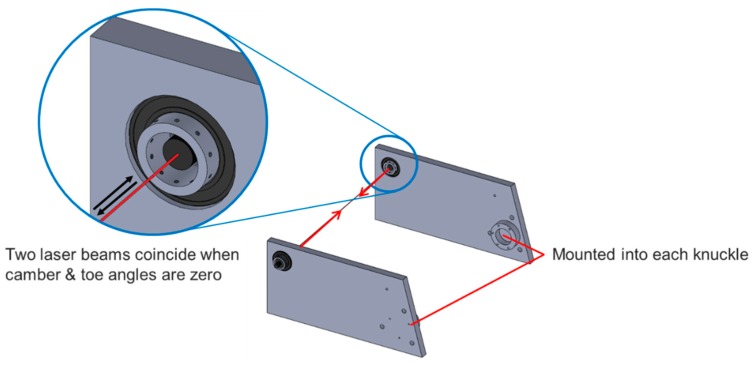
Zero camber and toe angles.

The proposed wheel alignment inspection employs laser modules. A pair of precisely manufactured jigs are mounted into both knuckles, as shown in Figure 5, and two laser modules are orthogonally mounted in the jigs. The laser modules face each other and, if the laser beams from two sources coincide with each other, it means that the camber and toe angles of both wheels are zero, as shown in Figure 6.

**Figure 7:**
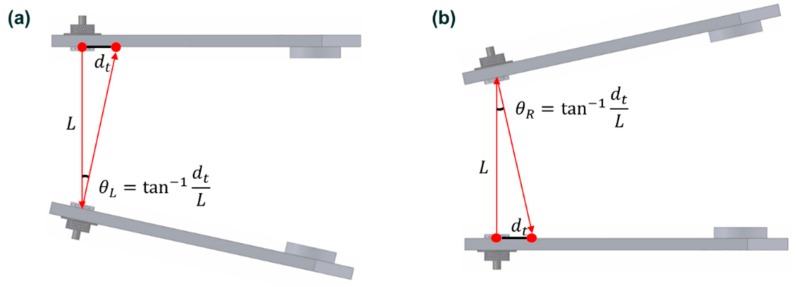
Toe angle measurement. (**a**) Left toe. (**b**) Right toe.

Figure 7 describes a toe angle measurement according to the proposed wheel alignment inspection. When a toe angle occurs at the left wheel, the laser module in the left wheel is horizontally steered and, accordingly, its laser mark on the right knuckle jig horizontally moves away from the right laser source. By measuring the horizontal distance between the source and the laser mark, the toe angle of the left wheel can be measured. The toe angle can be calculated using Equation (1).

(1) $$\theta =\tan^{-1}\frac{d_{t}}{L}$$

where $d_{t}$ is the horizontal distance between a laser source and the mark from the other laser source, L is wheel track width, and θ is right or left toe angle.

**Figure 8:**
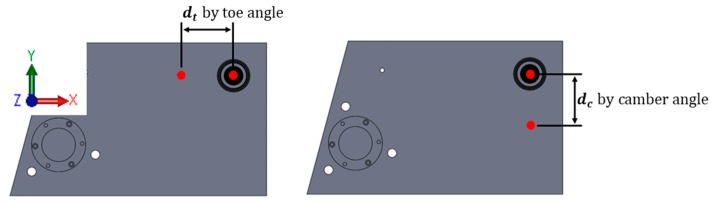
Direction of laser mark by toe and camber angles.

In the same manner, when a camber angle occurs at the left wheel, the laser module in the left wheel is vertically steered and, accordingly, the laser mark vertically moves away from the right laser source. By measuring the vertical distance between the source and laser mark, the camber angle can be obtained. The only difference between toe and camber angles is the direction of laser marks, as shown in Figure 8. The camber angle can be calculated using Equation (2).

(2) $$\varphi =\tan^{-1}\frac{d_{c}}{L}$$

where $d_{c}$ is the vertical distance between a laser source and the mark from the other laser source, and φ is left or right toe angle.

### 3.2. Laser Module Setup and Calibration

For accurate wheel alignment inspection, each laser module should be orthogonally mounted into each jig. If a laser module is not perfectly orthogonal to the jig, then measurement errors can result. Especially for toe angles whose setting range is usually from −0.2 to +0.2 degrees, the orthogonality of the laser modules is very crucial to the measurement results. In this study, a laser module with an adjustable beam size was chosen in order to prevent the measurement inaccuracies that can be caused by a large beam size. The minimum diameter of the beam is 1 mm which is adjusted by a screw head. The specifications of the laser module used in this study are shown in Table 1.

**Figure 9:**
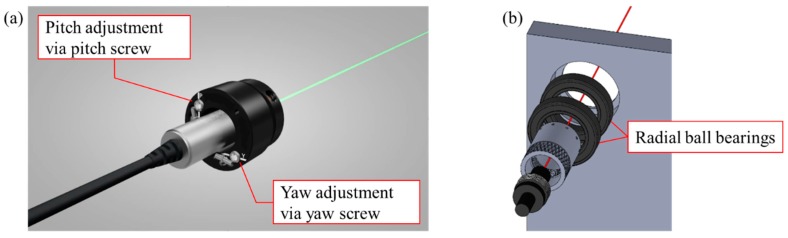
Laser module for calibration. (**a**) Pitch and yaw adjustment via screws. (**b**) Two radial ball bearings for rotating.

After the laser module is mounted in the jig, its yaw and pitch angles should be adjusted for complete orthogonality. This study used a kinematic adapter that was installed between the laser module and jig. This kinematic adapter is designed not only to facilitate the integration of the laser module in the jig, but also to provide pitch and yaw adjustments. By tightening or loosening the pitch and yaw screws (as shown in Figure 9a), the laser module can be completely orthogonal to the jig. In addition to the kinematic adapter, a pair of radial ball bearings were also used for the integration of the laser module, because the procedure of calibrating the laser module requires rotating the laser module with respect to the jig. By using two radial ball bearings in series (as shown in Figure 9b), the bearing rotational error caused by the bearing tolerance can be prevented.

**Figure 10:**
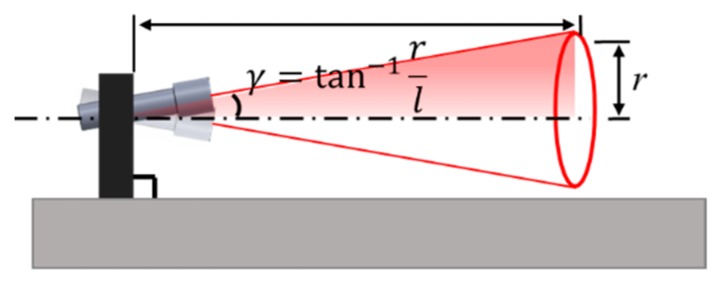
Orthogonal calibration of the laser module.

Figure 10 shows the laser module calibration procedure. A laser module is mounted in the jig and switched on. The laser beam is projected to the board at a far end and the laser mark creates a circle on the board as the laser module is rotated. The larger the orthogonal error is to the jig, the larger the circle is. By tightening or loosening the pitch and yaw screws, the circle can be decreased to its smallest possible size. The orthogonal error can be calculated by Equation (3).

(3) $$\gamma =\tan^{-1}\frac{r}{l}$$

where l is the distance from the laser module and a board, r is the diameter of the circle, and γ is the angle between the circle center and the laser module.

In this study, a laser beam was projected onto a board located 8000 mm away from the laser module, achieving a 0.02 degree orthogonal error. 

### 3.3. Experimental Setup

**Figure 11:**
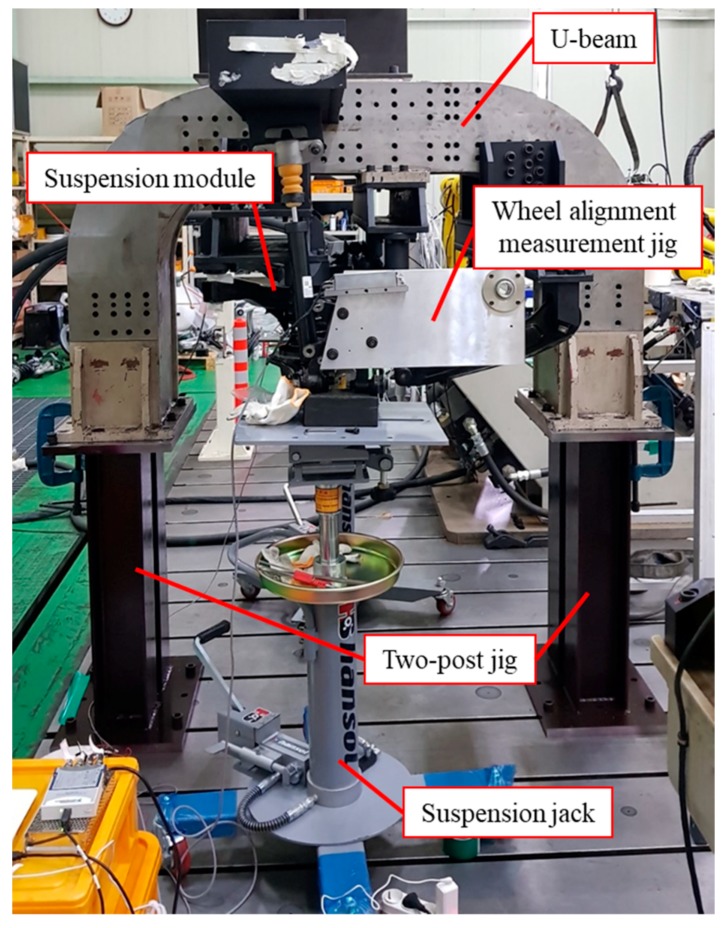
Experimental setup for wheel alignment inspection.

Figure 11 shows an experimental setup for the wheel alignment inspection using laser modules. A suspension module for the wheel alignment inspection is mounted onto jigs. The jigs are also screwed to a U-beam that is placed on two posts that are fixed on a surface table. The knuckle is linked to suspension components such as the lower arm, shock absorber, and trailing arm, and its kinematic motion is geometrically determined by the suspension components. Accordingly, the toe and camber angles of the suspension module vary with the vertical position of the knuckle. In this study, the knuckle was lifted to the vertical position predetermined by the automaker for accurate wheel alignment inspection, and two hydraulic suspension jacks were used to lift the knuckles. Two wheel alignment measurement jigs with the calibrated laser modules were mounted onto the knuckles.

Proper reflective surfaces have been employed in many studies using laser devices to perform measurements [17,18]. In this study, two laser-detecting cards were attached to each surface where the laser light was projected. These cards were photosensitive and enabled the easy location of ultra-violet (UV) and visible light beams and focal points.

## 4. Results & Verification

In order to verify the wheel alignment inspection proposed in this study, a new way of using a vision-based system with two cameras was also developed. As previously mentioned, the computer vision-based inspection system with four CCD cameras that is widely used in most automotive maintenance shops needs four wheel centers to measure the toe and camber angles. Accordingly, these cannot be applied during verification. In this study, two CMOS cameras and their accessories were adopted for verification.

A suspension module was designated as a target for verification. The target’s camber and toe angles were measured by the laser and camera modules, and the results were compared. The proposed wheel alignment method using laser modules measures camber and toe angles by measuring the horizontal and vertical distances of the laser marks. Accordingly, its sensitivity depends on the wheel track width of the suspension module. In the case of the target suspension module, the laser module’s sensitivity was 36.53 mm/degree, which means that if one degree shifts in the toe or camber angle (shown in Figure 8), then the laser mark moves 36.53 mm horizontally or vertically.

### 4.1. Verification System Configuration and Inspection Mechanism

**Figure 12:**
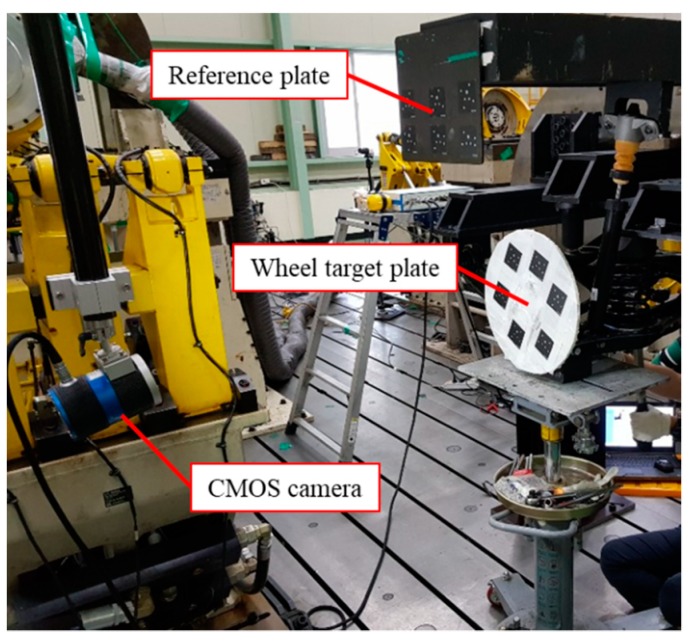
Verification system for wheel alignment inspection.

The two-camera optical system used for verification consists of two CMOS cameras, two reference plates, two wheel target plates, an image data acquisition device, a power source, and jigs. It was originally designed for measuring vehicle wheel movements, whereby each wheel was monitored by a camera capturing the wheel as well as its reference plate [15]. The specifications of the optical system are described in Table 2. The two wheel target plates are mounted into each knuckle of the suspension module, whereas the two reference plates are fixed by jigs. Each reference plate is positioned close to each wheel target plate, but usually in the front of the wheel target plate, as shown in Figure 12. The cameras are fixed at a distant position from the suspension module so that they can capture the wheel target and reference plates in one picture frame. These high resolution CMOS cameras can measure the toe and camber angles of the wheel target plates by detecting their relative motions with respect to the reference plates. For this measurement mechanism, some preparatory procedures are required.

The preparatory procedures consist of three steps: 3D space creation, absolute coordinate creation, and local coordinate creation and wheel alignment inspection.

(i) 3D Space Creation

**Figure 13:**
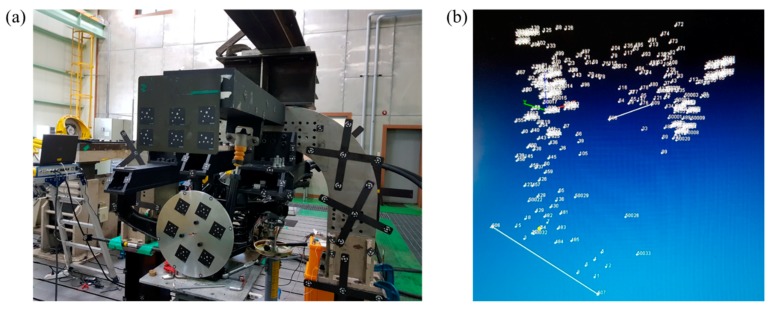
3D space creation. (**a**) Markers on the suspension module and jigs. (**b**) Created 3D space using markers.

There are many ways to create a 3D space using cameras [19,20,21]. In this study, 3D space creation with IR cameras and retro-reflective markers was employed. Each camera was equipped with an IR pass filter and a ring of IR LEDs around the lens, which periodically illuminated the measurement space with IR light. Retro-reflective markers, on the other hand, reflected the incoming IR light back to the cameras. The IR reflections were detected by the cameras, then internally processed by the optical software. This system calculates the 2D marker positions in image coordinates with high precision. In an optoelectronic system, a minimum of three markers are basically required to measure a rigid body [22], but for the system employed in this study, multiple markers were randomly placed onto the object in order to create the 3D space of the object. Figure 13a shows the markers placed on the suspension module and jigs for the 3D space creation. After sticking hundreds of markers onto the object, hundreds of photos were taken by a camera. These photos were sent to the host computer, then processed by the optical software to create the 3D space of the object. Figure 13b shows the created 3D space of the suspension module with retro-reflective markers and IR LED cameras.

(ii) Absolute Coordinate Creation

**Figure 14:**
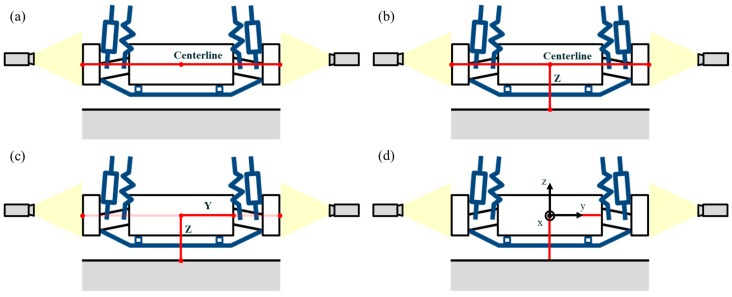
Absolute coordinate creation. (**a**) Centerline. (**b**) Z axis. (**c**) Y axis. (**d**) X axis.

Retro-reflective markers were also placed on the target wheel plates. In addition to the markers, there were single-dot-type markers along the rim of the target wheel plates, as shown in Figure 13a. By calculating the center of the single-dot-type markers on the target wheel plate, the positions of each wheel center can be defined in the 3D space. Figure 14 shows the procedures that were used to create the absolute coordinate of the suspension module. First, an absolute plane is required to create the absolute coordinate, and the ground surface is the absolute plane. Once the centers of each target wheel plate are defined, the line that connects these centers is also defined, as shown in Figure 14a. This line is defined as the Centerline. This Centerline also represents the center between the two wheel centers. The normal vector from the absolute plane (the surface table) to the center of Centerline is defined as the Z axis (Figure 14b). Ideally, the Z axis and Centerline are supposed to be orthogonal each other. However, they are not actually orthogonal due to the machining and assembly tolerance of the jigs. This is the reason that the line from the two wheel centers is defined as the Centerline and not the Y axis. The Centerline and the Z axis create one plane, and the Y axis can be defined on that plane. This Y axis is not only on the plane created by the Centerline and the Z axis, but is also perpendicular to the Z axis (Figure 14c). Once the Z and Y axes are defined, then the X axis is defined by the right-hand rule, as shown in Figure 14d [23]. The XYZ coordinate created by these procedures is the basic coordinate needed to create two local coordinates at each wheel.

(iii) Local Coordinate Creation and Wheel Alignment Inspection

**Figure 15:**
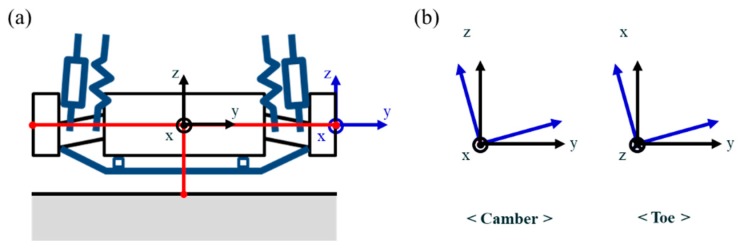
Local coordinate creation and wheel alignment inspection. (**a**) Local coordinate creation. (**b**) Camber and toe angle measurement.

Two local coordinates are created at each wheel by transferring the absolute coordinate to each wheel, and the toe and camber angles are obtained by the angular differences between the local coordinate and the target wheel plane, as shown in Figure 15a. Camber angles are the angular differences of the Y axis with respect to the X axis, and toe angles are the angular differences of the Y axis with respect to the Z axis, as shown in Figure 15b. 

### 4.2. Verification Results and Discussion

Verification tests were performed three times for each wheel, and the results are shown in Table 3. As mentioned earlier, toe angles of general passenger vehicles are usually set between −0.2 and 0.2 degrees, so the toe angles in the verification tests were also controlled in the same range. In the same manner, the camber angle was controlled between −2 and 2 degrees. For the entire verification tests, the maximum difference of the measurement values between the laser and camera modules was 0.071 degrees in the left camber angle, and the minimum difference was 0.007 degrees in the right camber angle. The mean value of the absolute difference was 0.027. Considering the cost and preparatory time difference between the two measurement methods, the proposed wheel alignment inspection in this study seems to offer very meaningful results.

The proposed wheel alignment inspection method uses two laser modules that are mounted into the wheel knuckles, and the toe and camber angles are determined by measuring the distance of the laser mark projected onto the opposite side of the laser mounting jig. Because of this measurement approach, when both wheels have non-null toe and camber angles, it can affect the measurement results. For instance, if the left wheel exhibits one degree of camber angle, then it basically has 36.53 mm of the laser mark distance on the right side of the knuckle plate. However, if the right wheel exhibits one degree of camber angle at the same time, then the laser mark distance of the left wheel becomes 36.534 mm, which is 1.00011 degrees. Therefore, this error can be negligible. Furthermore, the measurement results can also be affected by the manufacturing tolerances of the jigs. Especially for the knuckle jigs, the parallelism and perpendicularity tolerances may be critical to the measurement results. In this study, in order to prevent measurement errors, the parallelism and perpendicularity tolerances of the knuckle jigs were 0.01 mm and the surface roughness was 0.1 μm. 

## 5. Conclusions

This study proposes a novel method for vehicle wheel alignment inspection. Unlike the wheel alignment inspection for full cars, which makes use of four wheel centers to measure toe and camber angles, wheel alignment inspection of a suspension module in development requires a new approach, since one suspension module has only two wheel centers. This study employed two laser modules for inspecting the wheel alignment of suspension modules in development. Two laser modules were orthogonally mounted onto the jigs of the left and right wheel knuckles, and each beam from the two laser sources was projected onto the other jig plane. By measuring the horizontal and vertical distances of the laser marks from the laser sources, the toe and camber angles of each wheel plane were obtained. 

The measurement accuracy of the proposed wheel alignment inspection can be guaranteed only when perfect orthogonality between the jig and the laser module is achieved. For perfect orthogonality, the angle between the jig and the laser module was precisely adjusted by rotating the laser module. Using a laser module mounted on the jig and a plane board placed at the far end of the laser module, the laser beam mark drew a circle on the board as the laser module rotates. By measuring and minimizing the diameter of the circle, the orthogonality between the jig and laser module was adjusted. By means of adjustment, 0.02 degrees of orthogonality was achieved.

After setting up the wheel alignment conditions for a suspension module using several experimental parts such as a U-beam, surface table, pillars, and jigs, the wheel alignment inspection for the suspension module was performed. In this study, a vision system with two CMOS cameras was employed to verify the proposed wheel alignment inspection method. For the verification, two ultra-high resolution CMOS cameras were used and a 3D space were created by the cameras. In this 3D space, one absolute coordinate system from two wheel centers was created and two local coordinate systems at each wheel center were also created. By measuring the rotation angles between the absolute and local coordinate systems, the toe and camber angles of each wheel plane were obtained. From the verification results, it can be seen that the proposed wheel alignment inspection using laser modules can provide very accurate measurement results.

The wheel alignment inspection proposed in this study can not only give very reliable results but was also very quick and cost-efficient. Therefore, it can save time and cost for automakers.

## Figures and Tables

**Table 1:** Specifications of the laser module.

**Beam Shape (Collimated)**	Elliptical
**Beam Size**	ф 1.0 mm~5.0 mm
**Focused Spot Diameter**	30 μm
**Operating Voltage**	4.9 V~5.2 V
**Wavelength**	630 nm~645 nm
**Power**	4.0 mW~5.0 mW

**Table 2:** Specifications of camera system.

**Type of camera**	complementary metal-oxide semiconductor (CMOS)
**Acquisition frequency**	500 Hz
**Measurement range**	±45°
**Distance camera/wheel adapter**	0.5 m
**Position accuracy**	±0.1 mm
**Angular accuracy**	±0.015°

**Table 3:** Measurement and verification results.

			Laser (deg)	Camera (deg)	Difference (Laser-Camera) (deg)
Toe	1	Left	−0.15	−0.127	−0.023
		Right	−0.15	−0.158	0.008
	2	Left	0.01	−0.008	0.018
		Right	0.01	−0.013	0.023
	3	Left	0.16	0.187	−0.027
		Right	0.15	0.176	−0.026
Camber	1	Left	−1.45	−1.379	−0.071
		Right	−1.51	−1.482	−0.028
	2	Left	−0.10	−0.073	−0.027
		Right	0.05	0.043	0.007
	3	Left	1.50	1.461	0.039
		Right	1.45	1.482	−0.032

## Nomenclature

α	Angle between normal vectors of Plane A and B
β	Angle between centerline and normal vector of Plane A
θ	Toe angle (deg.)
d_t	Horizontal distance between laser source and the mark from the other laser source (m)
L	Wheel track width (m)
φ	Toe angle (deg.)
d_c	Vertical distance between laser source and the mark from the other laser source (m)
γ	Angle between circle center and laser module (deg.)
r	Diameter of circle drawn by laser mark (m)
l	Distance from laser module and board (m)
